# It Takes “Guts” to Cause Joint Inflammation: Role of Innate-Like T Cells

**DOI:** 10.3389/fimmu.2018.01489

**Published:** 2018-06-29

**Authors:** Céline Mortier, Srinath Govindarajan, Koen Venken, Dirk Elewaut

**Affiliations:** ^1^Department of Rheumatology, Ghent University Hospital, Ghent, Belgium; ^2^Unit for Molecular Immunology and Inflammation, VIB Center for Inflammation Research, Ghent University, Ghent, Belgium

**Keywords:** innate-like T cells, invariant natural killer T cells, mucosal-associated invariant T cells, CD1, MR1, rheumatic diseases, inflammatory bowel disease, gut–joint axis

## Abstract

Innate-like T cells such as invariant natural killer T (iNKT) cells and mucosal-associated T (MAIT) cells, characterized by a semi-invariant T cell receptor and restriction toward MHC-like molecules (CD1 and MR1 respectively), are a unique unconventional immune subset acting at the interface of innate and adaptive immunity. Highly represented at barrier sites and capable of rapidly producing substantial amounts of cytokines, they serve a pivotal role as first-line responders against microbial infections. In contrast, it was demonstrated that innate-like T cells can be skewed toward a predominant pro-inflammatory state and are consequently involved in a number of autoimmune and inflammatory diseases like inflammatory bowel diseases and rheumatic disorders, such as spondyloarthritis (SpA) and rheumatoid arthritis. Interestingly, there is link between gut and joint disease as they often co-incide and share certain aspects of the pathogenesis such as established genetic risk factors, a critical role for pro-inflammatory cytokines, such as TNF-α, IL-23, and IL-17 and therapeutic susceptibility. In this regard dysregulated IL-23/IL-17 responses appear to be crucial in both debilitating pathologies and innate-like T cells likely act as key player. In this review, we will explore the remarkable features of iNKT cells and MAIT cells, and discuss their contribution to immunity and combined gut–joint disease.

## Introduction

Over the past decades, innate-like T cells have gained increasing attention given their unique biology and potential involvement in multiple immune and inflammatory diseases. Those cells, with overlapping features of both the innate and adaptive immune system, are characterized by an antigen-specific semi-invariant T cell receptor (TCR) with restricted V(D)J rearrangement. Innate-like T cells are able to rapidly produce cytokines, which makes them an ideal first-line defense against microbial infections (1). However, it has become clear that these cells show functional plasticity and can be skewed toward a more pro-inflammatory state (2). Two members of this unconventional T cell population are invariant natural killer T (iNKT) cells and mucosal-associated invariant T (MAIT) cells. Both cell types have the unique feature of recognizing atypical non-peptide antigens presented by highly conserved MHC-related molecules, respectively CD1 and MR1. iNKT cells respond to glycolipid molecules, whereas MAIT cells can be activated by vitamin B2 (riboflavin) metabolites, which are intermediates from bacterial and yeast biosynthetic pathways (3). Gamma delta (γδ) T cells are a third innate-like T cell population (4), but the focus here will be on CD1- and MR1-restricted T cells. In this review, we want to highlight the intriguing nature of these cells and discuss what is known about their role in rheumatic diseases like spondyloarthritis (SpA) and rheumatoid arthritis (RA), next to inflammatory bowel diseases (IBD).

Spondyloarthritides are a group of chronic inflammatory disorders that primarily affect the musculoskeletal system and are multifactorial in origin. Some subtypes affect mainly the axial joints (spine and sacroiliac joints), with ankylosing spondylitis (AS) as prototypical disease, while others have a more peripheral manifestation (arthritis of the limbs and enthesitis) (5). RA, another chronic rheumatic disorder, has an autoimmune basis and is characterized by the presence of autoantibodies direct against i.a. citrullinated antigens (6). Most commonly involved are the small joints of hands and feet, often with a symmetrical distribution, whereas in SpA joint inflammation is generally non-symmetrical. IBD is the collective term for a group of inflammatory diseases of the digestive tract, leading to gastrointestinal complaints. The two best known subtypes are Crohn’s disease (CD) and ulcerative colitis (UC) (7).

Remarkably, SpA often is accompanied by extra-articular manifestations, such as acute anterior uveitis, psoriasis, or IBD. Histological evaluation showed that about 50% of SpA patients without gastrointestinal symptoms have microscopic intestinal inflammation (8), of which a fraction (5–10%) develops CD over time (8, 9). Furthermore the presence of subclinical gut inflammation is associated with shifts in the composition of the gut microbiome (10–12). A state of intestinal dysbiosis has also observed in RA and IBD patients (6, 11, 13–15). Additionally in RA, there is a significant correlation with periodontitis and the presence of *Porphyromonas gingivalis* in the oral cavity. This bacterium could play an important role in the pathogenesis of RA through citrullination of proteins using a specific enzyme (peptidyl arginine deiminase), potentially leading to the production of anti-cyclic citrullinated peptide (anti-CCP) autoantibodies relevant to RA disease (16, 17). This finding underscores that microorganisms can have a direct pathological role in disease pathogenesis. On the other hand, alterations in microbial composition can also play an indirect role by modulation of specific immune cell functions relevant for these diseases. Hence, the ability of recognizing bacterial antigens (or derived products) combined with their clear presence at barrier sites, makes innate-like T cells an appealing target to study in the context of the gut–joint axis in rheumatic diseases.

A crucial role for pro-inflammatory cytokines in the pathogenesis of SpA, RA, and IBD, is confirmed by current knowledge from genome-wide association studies (GWAS) and anti-cytokine trials. Interestingly, SpA, RA, and IBD share clinical responsiveness to anti-tumor necrosis factor (TNF)-α therapy but significantly differ in their response toward inhibition of other key inflammatory cytokines like IL-17. Over the years, the interleukin (IL)-23/IL-17 immune axis has manifested as a major player in the pathogenesis of SpA (18). GWAS studies have revealed polymorphisms in the *IL23R* gene associated with both SpA and IBD (19). Furthermore, there is extensive evidence from *in vivo* models, translational studies, and clinical trials (2, 20–22). Curiously, anti-IL-17 treatment was not effective in patients with RA or IBD with some reports even suggesting a worsening of IBD, which might be linked to an effect on barrier integrity (23–25). IL-23 is essential for the terminal differentiation and inflammatory functions of T helper-17 (Th17) cells. Interestingly, it has been shown that also innate-like T cells express the key Th17 transcription factor retinoic acid receptor-related orphan receptor-γt (RORγt) and that they can respond toward IL-23 by producing IL-17 and related cytokines like IL-22 (22). The importance of this finding was underscored by a mouse study, in which IL-23 overexpression (an SpA-like model using minicircle DNA technology) could induce enthesitis independent of conventional Th17 cells (26). As disease induction did require the presence of CD4^−^CD8^−^ T cells, there could be a role for IL-23 responsive innate-like T cells (27).

## iNKT Cells

### Biology and Localization

Invariant natural killer T cells are CD1d-restricted T cells which express a semi-invariant TCR consisting of an invariant α chain [in particular, the variable (V) and joining (J) segments Vα14–Jα18 in mice and Vα24–Jα18 in humans], combined with a restricted β chain repertoire, usually Vβ2, Vβ7, or Vβ8.2 in mice and Vβ11 in humans (28, 29). Identification of these cells in mice can be performed by the use of CD1d tetramers and in humans by using CD1d tetramers, a specific Vα24Jα18 Ab (clone 6B11) or the combination of anti-Vα24 and anti-Vβ11 antibodies. In contrast to conventional T cells which detect self or foreign peptide antigen–MHC complexes, iNKT cells recognize only glycolipid antigens bound to CD1d, a MHC class I-like glycoprotein (30). Currently, identified antigens are predominantly of non-mammalian nature, with α-galactosylceramide (α-GalCer) as the most potent and best studied example. However, also microbial derived (31) and endogenous ligands have been described (28, 32, 33). Of note, the human genome encodes five CD1 genes (CD1a, b, c, d, and e) whereas only CD1d is expressed in mice, and human CD1a, b, and c restricted T cells have been described too (34).

A hallmark of iNKT cell biology is the ability to secrete large amounts of cytokines and chemokines upon TCR recognition of lipid antigen–CD1 complexes or *via* indirect (TCR independent, mainly cytokine driven) stimulation, hereby acting as a “bridge” between innate and adaptive immune responses (35, 36). In analogy to classification of conventional T cells based on their cytokine production, iNKT cells can be subdivided in NKT1, NKT2, and NKT17 cells (37). Each of these subsets expresses distinct transcription factors which correlate with their capacity to secrete specific cytokines. NKT1 cells are T box transcription factor TBX21 (T-bet) positive and primarily secrete interferon (IFN)-γ, NKT2 cells express high levels of GATA-binding protein 3 (GATA3) and promyelocytic leukemia zinc finger protein (PLZF), and produce IL-4 and IL-13, and NKT17 cells express RORγt next to intermediate levels of PLZF and produce IL-17 as signature cytokine (38–40). All these subsets acquire their functional capacity during the development in the thymus and are distributed to the peripheral organs in a tissue-specific manner (41). However, there are also reports suggesting that peripheral iNKT cells are able to further functionally differentiate under inflammatory conditions (42, 43). In addition, it is also clear that iNKT cells experience further maturation at mucosal surfaces (e.g., lung and gut) as evidenced from experiments with germ-free mice (44, 45).

Finally, next to above-mentioned subsets, also other particular iNKT cells, such as NKT_reg_ (FOXP3^+^) (46), NKT_FH_ (CXCR5^+^PD-1^hi^) (47), NKT10 cells (48), and adipose tissue residing iNKT (PLZF-E4BP4^+^) cells (49) have been described and warrant further investigation. The frequency of iNKT cells in mice is substantially higher compared to humans. The majority of murine iNKT cells are found in the liver (20–40%), whereas iNKT cells constitute only 1% of cells in the human liver. Moreover, the iNKT cell frequencies in human peripheral blood samples shows significant inter-donor variation (approximately 0.01–0.5% of T cells) which makes the study of human iNKT cell biology more challenging.

### Contribution to Gut and Joint Disease

Considering the ability of iNKT cells to produce copious amounts of immunomodulatory cytokines, several studies have assessed the capacity of iNKT cells to modulate autoimmune diseases (50–54). Some have shown that activation of iNKT cells can protect from joint inflammation, while others mentioned exacerbation of disease (2, 35, 52–58). In TNF^ΔARE/+^ mice, a TNF-driven SpA-like animal model for combined gut and joint inflammation, iNKT cells can dampen arthritis and ileitis by producing immunomodulatory cytokines after activation by TNF-driven CD1d^high^ dendritic cells (DCs). Interestingly, the frequency of the latter cell population is increased in synovial fluid from SpA patients (52). This example, next to evidence from an iNKT cell-dependent infectious disease *in vivo* model, suggests that inflammatory DCs can pick up antigens from the microbiota or microbial-derived products at the intestinal draining sites and subsequently activate iNKT cells. Furthermore, the crosstalk between DCs and iNKT cells was found to be TNF-mediated (52, 59, 60). Collagen-induced arthritis (CIA) and collagen antibody-induced arthritis (CAIA), two mouse models for RA, have revealed contradictive results. While several reports suggested a pathogenic role (55, 56, 61, 62), iNKT cells protected from disease in a number of studies (54, 63, 64). Conflicting outcomes could originate from differences in the stimulating ligand and the time point of iNKT cell activation, since these appeared to be crucial factors (54). Regarding human joint disease, it has been described that RA patients have lower frequencies of both CD4^−^ and CD4^+^ iNKT cells in peripheral blood compared to healthy controls, and they were skewed toward a Th1 phenotype (65–67) and a more restricted iNKT-TCR repertoire (68). There is no clear information regarding iNKT cell function in SpA disease so it will be of interest to study these, but also other innate-like T cells, in the context of joint–gut pathology in SpA patients.

Similar to joint disease, dichotomous effects of iNKT cells were observed for IBD (69). In dextran sodium sulfate-induced colitis, a model for human UC, activation of iNKT cells by α-GalCer ameliorated disease (70, 71). Adoptive transfer in iNKT deficient mice also had a protective role (70, 72, 73). In contrast, iNKT cells exacerbated inflammation in oxazolone-induced colitis, another UC model, as shown from results in CD1d and iNKT-deficient mice (74). Again it is clear that iNKT cells are involved in the pathogenesis, possibly even serving a dual role depending on the type of IBD (UC versus CD like) and the exact conditions of activation and further research is warranted to elucidate the mechanisms, ideally by using CD1d tetramer stainings. A large cohort of IBD patients showed that iNKT cells were decreased in the blood in both CD and UC compared to healthy individuals (75). The intestinal lamina propria of UC patients was found to have a strong abundance of type 2 iNKT cells that produced high amounts of the cytotoxic cytokine IL-13 (76, 77). Further studies are required to understand whether these disturbances in cell numbers in patients are a result of disease or whether iNKT cells are involved in development or persistence of inflammatory gut and joint disorders.

## MAIT Cells

### Biology and Localization

Mucosal-associated invariant T cells are an evolutionarily highly conserved cell population with two defining traits: the expression of a semi-invariant TCR and restriction of recognizing antigens presented by the MHC class I-related molecule MR1. Similar to iNKT cells, their TCR consists of an invariant TCR α chain paired with a limited array of Vβ chains (Vα7.2Jα33 paired with Vβ2 or Vβ13 in humans and Vα19Jα33 paired with Vβ6 and Vβ8 chains in mice) (3). Also, MAIT cells can be stimulated by both TCR-activation and TCR-independent signals, such as IL-18 (36, 78). In both humans and mice, the majority of MAIT cells in peripheral blood and tissues are CD4^−^CD8^−^ or CD8^+^ (in particular, more CD8αα than CD8αβ), besides very few CD4-expressing cells (79). The development occurs in the thymus, followed by an extrathymically maturation, a process that is regulated by multiple factors, including MR1, commensal gut microbiota, and the transcription factor PLZF (80), as illustrated by their absence in MR1-deficient and germ-free mice (81) and their severely reduced frequency in PLZF-deficient mice (82).

Identifying MAIT cells in human blood and tissue can be based on expression of TCR Vα7.2 (TRAV1-2) combined with the NK cell receptor CD161 and/or IL-18Rα (CD218). However, some of these surrogate markers are not present throughout the whole ontogeny, which has challenged accurately defining the cells. Recently, the production of MR1 tetramers meant a major revolution in this field, enabling the specific detection of MAIT cells in both humans and mice (82, 83). This has led to increased understanding of the development, which in mice occurs in three stages with only stage 3 being functionally competent. This model is largely in parallel with the development in humans (80). Furthermore, MR1 tetramers have allowed to describe different subsets within the MAIT cell population (84).

Mucosal-associated invariant T cells are predominantly found at mucosal and epithelial barrier sites. They are most abundant in the gastrointestinal tract and associated organs, such as mesenteric lymph nodes and the liver (in the latter organ representing 20–45% of all human T cells), but can also be found in the blood (1–8% of all human T cells). However, a lot of variation exists in the frequency of MAIT cells among humans, with age as an important determining factor (84). In mice, MAIT cells have a much lower frequency (at least 10-fold less than in humans) but are also mainly found at mucosal surfaces (82, 85). Because of their localization in close contact with the microbiota, it is believed that MAIT cells serve an essential role in modulating host-microbial interplay (81). They recognize and can be activated by vitamin B2 (riboflavin) metabolites, such as ribityllumazines [for example, 5-OP-RU or 5-(2-oxopropylideamino)-6-D-ribitylaminouracil] and pyrimidines (80). As many vitamin biosynthetic pathways are restricted to bacteria and yeasts, it is believed that MAIT cells detect these antigens to respond toward microbial challenges.

The majority of MAIT cells (>80%) in peripheral blood of healthy humans was found to produce the Th1-related cytokines interferon-gamma (IFN-γ) and TNF (80). Only a small fraction could produce IL-17A, consistent with a low expression of RORγt in healthy subjects. However, it seems that peripheral expansion and maturation is particularly important in human MAIT cells, illustrated by a dominant IL-17A-producing MAIT cell population in the liver (86).

### Contribution to Gut and Joint Disease

In contrast with their role as first-line responders against microbial infections (87), MAIT cells are also thought to be involved in a number of inflammatory and autoimmune disorders. In many of these diseases, a reduced systemic MAIT cell frequency compared with healthy individuals was observed, together with an increased abundance at sites where inflammation occurred (88, 89). For instance, IBD patients were found to have decreased peripheral blood MAIT cells with an enrichment in inflamed intestinal tissue (90, 91). In both RA (92) and AS (93, 94), there was a systemic decrease in MAIT cells accompanied by elevated cell numbers in the synovial fluid. It should be noted that in some of these diseases, like IBD and RA but not AS, results could be confounded by the use of corticosteroids as this has been associated with lower systemic MAIT cell frequencies (88). Furthermore, the identification in these studies was based on the expression of surrogate markers (TCRVα7.2^+^CD161^hi^) and not MR1-tetramer stainings. Upon activation, CD161 can be downregulated on MAIT cells, which could also have influenced these results (88).

Next to changes in frequencies, there were also phenotypical alterations in these diseases. In IBD, MAIT cells expressed higher levels of activation markers such as CD69 and they produced more IL-17 (90, 91). UC patients showed increased IL-18 serum levels and interestingly, a correlation was found with CD69 expression, suggesting that induced IL-18 secretion could have a role in activation of MAIT cells in these patients (91). The activation status of MAIT cells was positively correlated with disease activity of AS patients (94). An upregulation of IL-17 in these cells could also be observed in peripheral blood of AS patients compared to healthy controls, together with a lower IFNγ production (93, 94). Curiously, the higher proportion of IL-17^+^ MAIT cells in AS was only seen in male patients, while no differences in other clinical parameters existed. Another important finding was that MAIT cells in synovial fluid from AS patients show even higher IL-17 levels than in peripheral blood (93). These results support the idea that MAIT cells can contribute to inflammatory diseases in both the gut and joint. Interestingly, an elevated IL-17 production by MAIT cells was not found in RA neither in peripheral blood nor in synovial fluid (93), suggesting a differential mechanism in RA and AS disease. An important role could be attributed to IL-7 as gut and joint tissues of AS patients contained higher IL-7 levels than healthy controls, next to a higher IL-7R expression in blood-derived MAIT cells from AS patients. Furthermore, IL-7 priming induced IL-17 production by MAIT cells and, even more interesting, this response was substantially higher in AS patients (93). Anti-TNF therapy did not affect the MAIT cell number nor did it decrease production of IL-17 or IFNγ by MAIT cells, further underscoring the IL-23/IL-17 axis in innate-like T cells as a potential therapeutic target (94).

An effector role for MAIT cells in arthritis was demonstrated in MR1-deficient mice, after both CIA and CAIA disease induction. MR1 deficiency significantly reduced arthritis and adoptive transfer of MAIT cells to MR1^−/−^ mice exacerbated arthritis in CAIA (95). The situation in IBD is less clear, with one report showing that adoptive transfer of MAIT cells in mice with TNBS-induced colitis resulted in milder disease (96). However, caution should be taken in interpreting this result, since the identification of MAIT cells in which study was only based on Jα33 TCR, meaning that also non-MAIT conventional T cells were included (88).

## Conclusion

**Figure 1:**
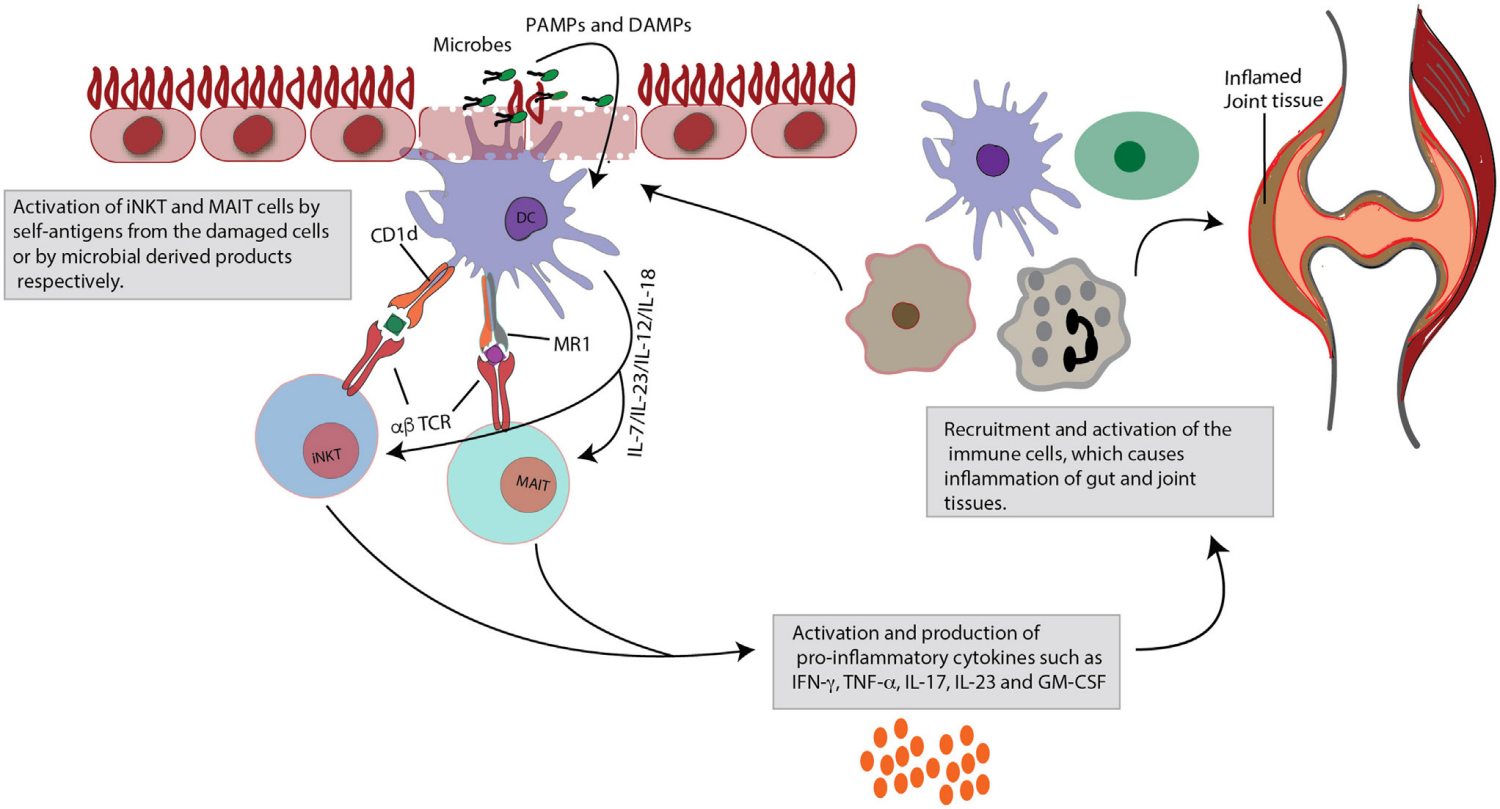
Role of invariant natural killer T (iNKT) and mucosal-associated invariant T (MAIT) cells in gut and joint inflammation. Upon activation, iNKT cells and MAIT cells rapidly produce large quantities of cytokines. This figure focuses specifically on their pro-inflammatory effects. iNKT and MAIT cells are characterized by a semi-invariant T cell receptor (TCR), which can detect ligands of bacterial or self-origin. These antigens are presented by monomorphic MHC class I-like molecules (CD1d for iNKT cells and MR1 for MAIT cells), expressed on dendritic cells (DCs). Next to this TCR-dependent stimulation, the cells can also be activated in a TCR-independent cytokine-induced manner, that is danger-associated molecular pattern (DAMP) or pathogen-associated molecular pattern (PAMP) mediated. Activation is quickly followed by production of pro-inflammatory cytokines, such as tumor necrosis factor (TNF)-α, interferon (INF)-γ, interleukin (IL)-23, IL-17, and granulocyte-macrophage colony-stimulating factor (GM-CSF). Subsequently, these mediators will recruit and activate other immune cells, which may contribute to inflammatory diseases, such as spondyloarthritis (SpA), characterized by combined gut and joint inflammation. Contribution to inflammation may arise from impaired regulatory capacities (e.g., iNKT cells) or through skewing to pro-inflammatory profiles (e.g., IL-17).

After believing for a long time that solely MHC–peptide complexes can be recognized by T cells, it is now known that TCRs can also bind (glyco)lipid, vitamin metabolites, and other non-peptidic antigens. These cell types include iNKT and MAIT cells, restricted to MHC class I-related molecules CD1d and MR1, respectively. Their evolutionary highly conserved nature indicates a strong selective pressure to be maintained in immune responses. Showing features of both the innate and adaptive immunity, these innate-like T cells act at the interface of the two systems. In this regard, it is not surprising that these cells, next to their distinct but still unclear resident role in liver tissue, are predominantly found at mucosal barriers, i.e., at sites where there is a close encounter with microorganisms. Next to direct activation by recognizing microbial-derived ligands *via* their semi-invariant TCR, they can also be activated indirectly (e.g., by cytokine and TLR-mediated signaling), upon which they respond by rapidly producing copious amounts of effector molecules as a first-line defense making them excellent gatekeepers against potential invasive pathogens (36, 97). However, innate-like T cells show a dichotomous phenotype, being not only protective but they are also thought to be involved in a number of immune and inflammatory diseases. Indeed, iNKT and MAIT cells might be skewed toward a predominant pro-inflammatory state in which secretion of key pathogenic cytokines such as IL-17 can cause tissue pathology (Figure 1) (26, 27). A pathological role for innate-like T cells is supported by evidence from diverse experimental models, although some conflicting results might reflect an aberrant role depending on the disease phenotype, activation kinetics, and the background of the animals (52, 57, 95). Interestingly, SpA-like gut and joint pathology shown in TNF- and IL-23-dependent animal models, such SKG and TNF^ΔARE/+^ mice does not develop under germ-free conditions (98, 99). This underscores the relevance of the host (immune)-microbial interplay in the induction of SpA-like disease features. Additionally, a state of dysbiosis as discovered recently in SpA patients (10–12) might contribute to chronicity of disease by e.g., dysregulating immunomodulatory T cell function and cytokine (TNF and IL-17) mediated responses. Although one has to keep in mind that a causal relationship has not been proven yet, we would postulate that it takes "guts" to cause joint inflammation as observed in SpA pathology. However, many questions still need to be addressed. For example, a specific role for innate-like T cells in these microbiota-mediated pathological events clearly awaits further investigation, especially in light of the complexity of the human disease. Future in depth immunoprofiling of innate-like Tcells, next to other immunomodulatory cells, in gut and joint tissues from SpA patients, combined with further exploration of their function and role in experimental models under different microbial conditions (e.g., conventional versus germ-free housing), will shed additional light on the precise nature of the relationship between these unconventional cell populations and the microbiota, and their contribution to gut and joint diseases.

## Author Contributions

CM and SG contributed equally to this work. KV and DE shared supervision.

## Conflict of Interest Statement

The authors declare that the research was conducted in the absence of any commercial or financial relationships that could be construed as a potential conflict of interest.
